# An Affectionate Lion

**Published:** 1892-01

**Authors:** 


### AN AFFECTIONATE LION.

The superintendent of the animal department at Woodward Garden, San Francisco, tells an interesting story of a lion which was at one time in the Garden.

At first the lion was so wild and fierce that it was dangerous to venture near his cage, but by persistent gentleness and kindness, the superintendent gradually made the beast grow so fond of him that it permitted him to go into the cage, and if he laid down beside it, the lion would raise its head, so as to give him a soft place to lie.

One day a drunken sailor came into the Garden and began teasing the lion. The superintendent went to the man and told him not to disturb the animals. The sailor, angered at the reproof, replied that he would do as he chose about it, and doubling up his fist, struck at the superintendent.

The lion upon this became frantic with rage. It roared fearfully, and dashed so violently against the bars of its cage that the sailor ran away in terror. Then the beast became quiet, and manifested the greatest delight when the superintendent went to it and caressed its head.

At length the lion became affected with a tumor. One or two slight operations had to be performed, and nobody could get near the beast except this one man. The lion let him cut, and looked at him gratefully all the time, licking his hand when it was over. The tumor grew, and a difficult operation had to be performed. It was with some apprehension that the superintendent undertook it, for the lion was restless with pain and discomfort. The doctor thought it unadvisable to administer ether. The physician drew a diagram of the operation, showing him where to cut. He followed their directions, talking soothingly to the noble beast the while, and the lion bore the knife bravely.

This operation afforded but temporary relief, and the poor beast began to suffer such pain that it was decided to kill it. The superintendent took his revolver, and after petting the animal, fired one shot through its head, putting the muzzle close to it. The lion gave him a pathetic look, in which there seemed to be a mixture of surprise and reproach, but no anger. It took three shots to kill it, and all the time the beast never took his eye off the man. The superintendent told the story with tears in his eyes.—*Ex.*

